# Single-cell genomics-based immune and disease monitoring in blood malignancies

**DOI:** 10.46989/001c.117961

**Published:** 2024-06-14

**Authors:** Anja C. Rathgeber, Leif S. Ludwig, Livius Penter

**Affiliations:** 1 Berlin Institute for Medical Systems Biology Max Delbrück Center for Molecular Medicine; 2 Department of Hematology, Oncology, and Tumorimmunology Charité - Universitätsmedizin Berlin; 3 Berlin Institute of Health at Charité - Universitätsmedizin Berlin; 4 BIH Biomedical Innovation Academy Berlin Institute of Health at Charité - Universitätsmedizin Berlin

**Keywords:** single cell sequencing, immune monitoring, immune checkpoint blockade, CAR T cell therapy, allogeneic hematopoietic stem cell transplantation, natural barcodes

## Abstract

Achieving long-term disease control using therapeutic immunomodulation is a long-standing concept with a strong tradition in blood malignancies. Besides allogeneic hematopoietic stem cell transplantation that continues to provide potentially curative treatment for otherwise challenging diagnoses, recent years have seen impressive progress in immunotherapies for leukemias and lymphomas with immune checkpoint blockade, bispecific monoclonal antibodies, and CAR T cell therapies. Despite their success, non-response, relapse, and immune toxicities remain frequent, thus prioritizing the elucidation of the underlying mechanisms and identifying predictive biomarkers. The increasing availability of single-cell genomic tools now provides a system’s immunology view to resolve the molecular and cellular mechanisms of immunotherapies at unprecedented resolution. Here, we review recent studies that leverage these technological advancements for tracking immune responses, the emergence of immune resistance, and toxicities. As single-cell immune monitoring tools evolve and become more accessible, we expect their wide adoption for routine clinical applications to catalyze more precise therapeutic steering of personal immune responses.

## Introduction

Blood malignancies have long been at the forefront of the development of immunotherapies. Spanning from the first complete remissions achieved with allogeneic hematopoietic stem cell transplantation (HSCT)^1,2^ to the most recent successes with chimeric antigen receptor (CAR) T cell therapies,^3,4^ bispecific T cell engagers (BiTEs)^5,6^ or immune checkpoint blockade (ICB),^7^ the field continuously pioneers strategies for therapeutic immunomodulation.^8–12^ These discoveries have been paralleled by an equally dynamic development of approaches to classify, quantify, and surveil immune cell function, including their impact on the immunomodulation of malignant cell populations. These efforts are thereby also driven by the necessity to confidently monitor these immunological interventions, to identify modulating factors that contribute to their clinical activity or resistance and, likewise, to precisely steer individual therapies.

This has led to a diverse repertoire of monitoring tools that enable tracking of immune cell function at high resolution, including flow cytometry,^13^ mass cytometry,^14^ immune receptor repertoire sequencing,^15,16^ donor chimerism tracking^17^ or cytokine profiling.^18^ It has also motivated the expansion of sequencing tool kits to identify and track the genetics,^19–21^ epigenetics^22,23^ or transcriptional states^24,25^ of malignant cell populations in response to immunotherapies.

With the maturation of single-cell sequencing technologies and their ability to measure multiple classes of molecular features at the same time,^26,27^ the prospect of a unified single-cell immune and cancer monitoring assay to integrate many of these tools is increasingly within reach. While associated costs and the complexity of single-cell analysis remain relatively high, great strides have been made that foreshadow the routine application of single-cell genomics for unprecedented immune and disease monitoring in blood malignancies.

## Approaches for immune and blood cancer cell monitoring

One of the main objectives for therapeutic monitoring of immune and malignant populations is to obtain real-time information on immunologic and cancer cell states to guide individual treatment decisions. An illustrative example is the serial quantification of donor chimerism and measurable residual disease (MRD) in the post-transplant course to steer immunosuppression and initiate early immunotherapeutic interventions like donor lymphocyte infusion (DLI) when an incipient relapse arises.^28–31^

**Figure 1.**
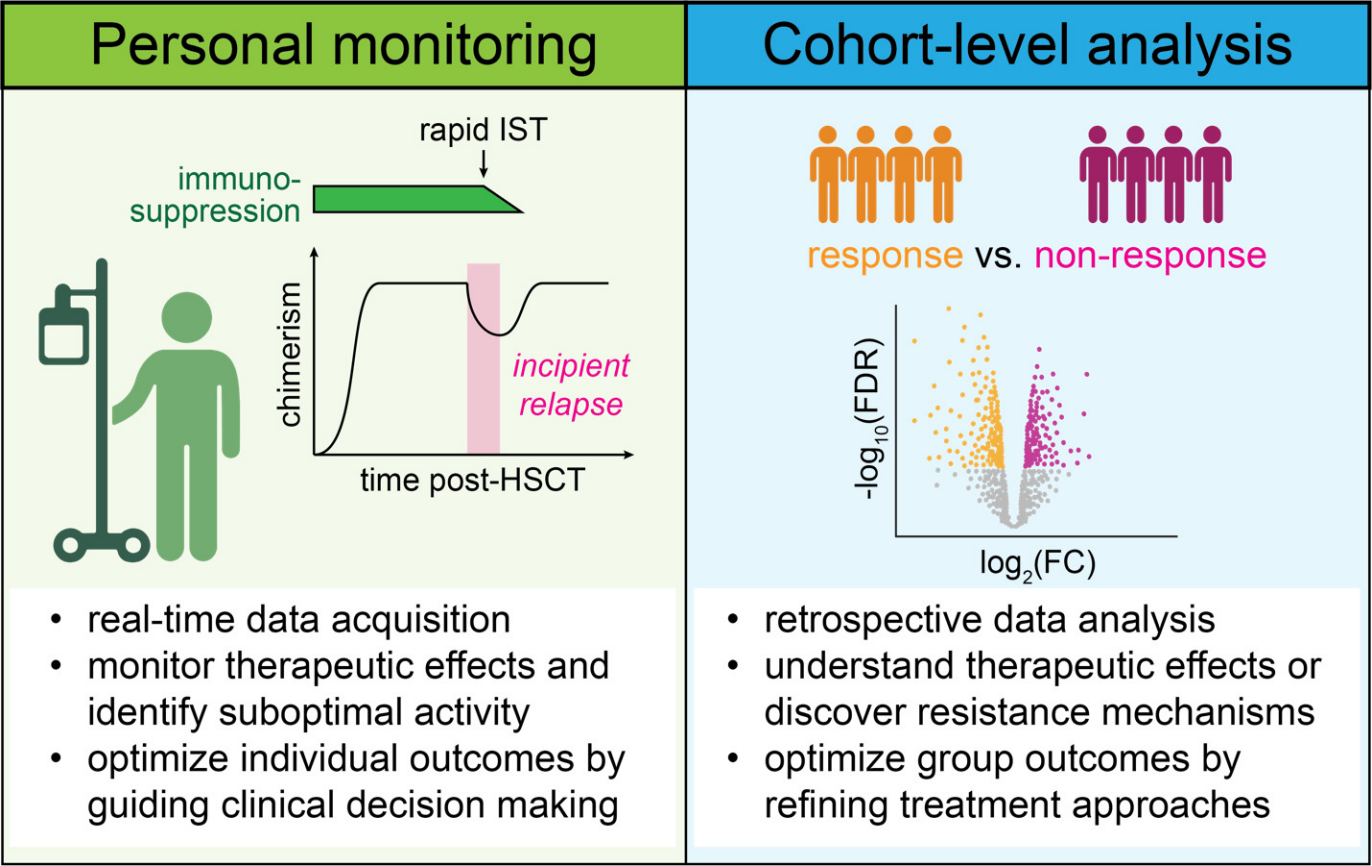
Individual and cohort-level immune monitoring. Real-time monitoring of immune cell responses provides opportunities to monitor therapeutic effects and to detect the need for additional interventions to optimize an individual treatment approach. One such example is rapid immunosuppression tapering (IST) in the wake of worsening donor chimerism and incipient relapse (left). Retrospective cohort-level analyses are performed on banked sample collections to discover predictive biomarkers, pharmacodynamics, and mechanisms that drive responses, resistance, or emergence of immune toxicities to improve treatment approaches for future cohorts (right).

At the cohort level, immune monitoring is used to gain deeper insights into the biology of immunomodulatory treatments through retrospective analyses of banked samples, to improve therapeutic efforts for future cohorts. For these systematic studies, four broad areas of research include i) the identification of predictive biomarkers for successful treatment or occurrence of adverse events, ii) elucidation of effects on immune cell populations induced by the treatment, irrespective of response (i.e., pharmacodynamics), iii) dissection of specific mechanisms that lead to responses or immune toxicities and iv) the discovery of processes that contribute to therapeutic resistance (**Fig. 1**).

Most of these questions entail aspects of immune cell function and responses in cancer cells in a constant feedback loop and make their integrative analysis desirable. However, immune and malignant cells are often studied individually for practical reasons. This is an opportunity for single-cell technologies to improve current research approaches, as they can dissect molecular and cellular mechanisms at multiple levels from the same sample and dataset.

### Immune cells

Immune function results from a highly dynamic and complex network of dozens of different cell types with complementary functions. Comprehensively monitoring these systems, therefore, requires obtaining information at several organizational levels, including i) immune cell type identity of the major cell lineages and their subsets, ii) the state and functional capacity of individual cells within these subsets, iii) immune cell receptor repertoires as the basis for antigen recognition, including T cell,^32–34^ B cell^35^ or Killer Ig-Like receptors^36^ (TCR, BCR, KIR), and iv) the environmental milieu immune cells exercise their function in, which may be modulated by direct interactions with adjacent cells or through indirect cellular communication (i.e., cytokines). Early single-cell immune phenotypic studies based their cell type annotation largely on the identification of gene expression clusters, which proved challenging to link to established flow cytometric definitions.^37^ This is increasingly overcome by utilizing single-cell atlases^38–40^ that provide a common framework for the annotation of single-cell RNA sequencing (scRNA-seq) profiles, or integration methods that enable cross-study comparison of single-cell datasets^41–43^ and allow the placing of single-cell sequencing results into context with flow cytometric data.

The introduction of 5’ scRNA-seq technologies in combination with the detection of oligonucleotide-conjugated antibodies (i.e., cellular indexing of transcriptomes and epitopes – ‘CITE-seq’)^44,45^ has provided a platform that enables to resolve most of these immunogenomic features with one assay. It is now routinely possible to obtain BCR and TCR repertoires of thousands of single cells, and most recently emerging technologies enable the identification of antigen-specific T cells directly through integration of tetra- and dextramer methods with scRNA-seq.^46,47^ Finally, the nascent field of spatial transcriptomics is starting to provide direct insight into immune cell interactions and higher levels of topical and neighborhood organization.^48^

### Cancer cells

Surveillance of malignant cell populations follows three main questions that include i) the classification of malignant cells, ii) their sensitive identification, for example in the form of MRD and iii) tracking of genetic/molecular changes within the malignant populations during therapeutic pressure such as their clonal evolution or the emergence of specific somatic mutations that convey resistance to a given treatment.^19,49^ To some extent, these questions can be addressed with flow cytometry (i.e., phenotyping and MRD detection) through identifying leukemia-associated immune phenotypes (LAIP), but the most versatile and unambiguous approach is the detection of genetic markers through sequencing. The advantage of genetic markers (somatic nuclear DNA mutations, chromosomal copy number aberrations, and more recently mitochondrial DNA mutations^50^) is that they can be utilized for all three aspects of tumor cell monitoring and that their binary property (presence or absence) is highly accessible to automated computational analysis.

For the detection of such natural genetic barcodes with single-cell sequencing, the choices are DNA- and RNA-based platforms which come with their individual strengths and advantages.^51^ In general, DNA-based single-cell sequencing, especially amplicon-based strategies, can obtain genetic information in thousands of cells and may be combined with protein detection that can distinguish immune and cancer cell populations.^20,52^ On the other hand, RNA-based detection of natural barcodes is more challenging, and has varying degrees of success, as it is dependent on the expression levels of genes that harbor a genetic marker or are part of a region with structural chromosomal changes.^53–56^ Nevertheless, whole-transcriptome single-cell RNA sequencing has the advantage that it can also provide detailed information on other cellular characteristics such as transcriptionally defined cell states that cannot be resolved with surface marker expression or immune receptor sequences, which are currently not easily accessible with DNA-based sequencing. A potential solution is sequencing platforms that provide information on DNA- and RNA properties, which are currently still associated with very high costs, lower data quality compared to isolated DNA or RNA sequencing, and, often, lower throughput.

A growing number of clinical studies that have leveraged single-cell genomics platforms for immune cell monitoring evidence the strength of providing a multi-layered read-out of immune cell properties and blood cancer dynamics following therapeutic interventions.

## Allogeneic HSCT

**Figure 2.**
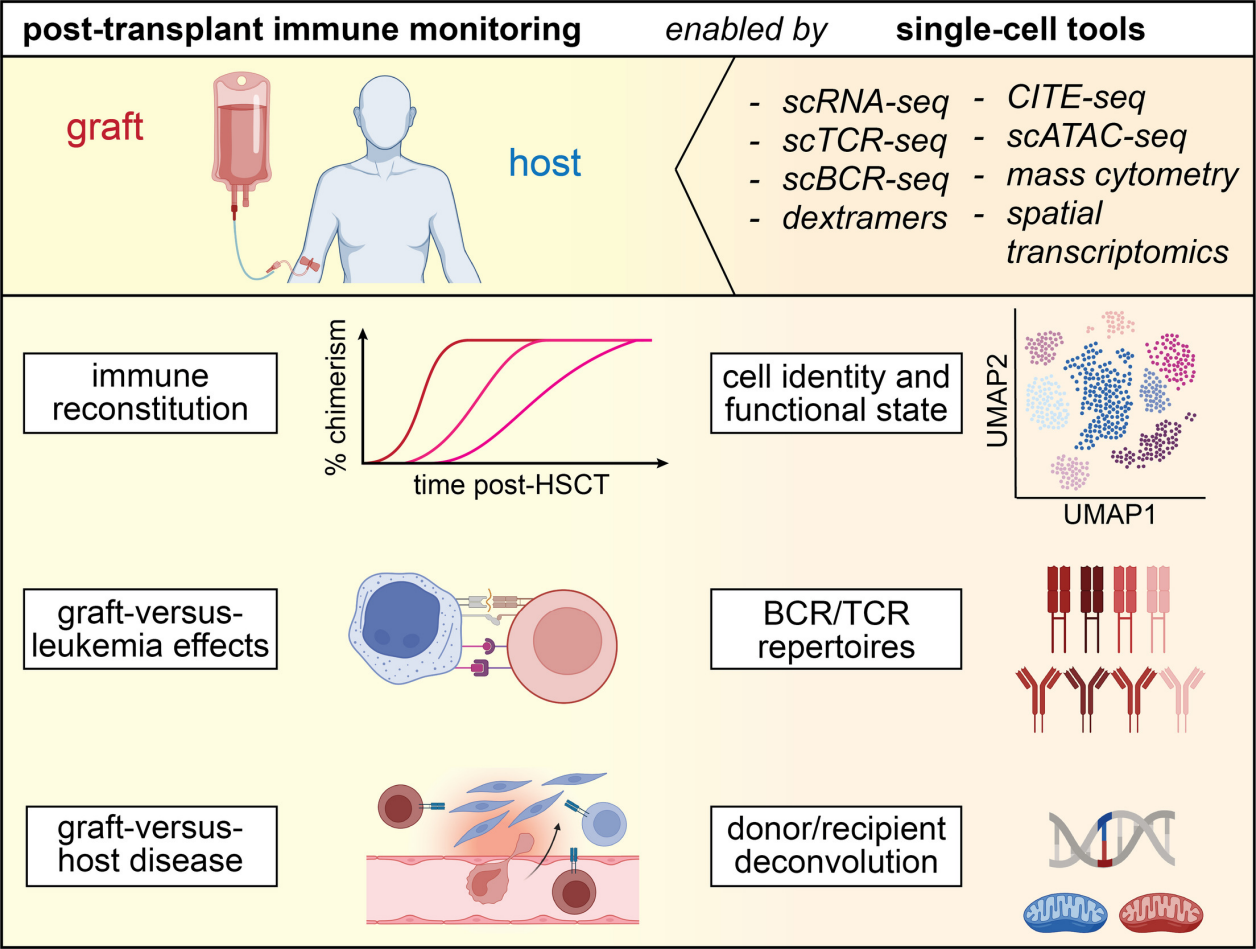
Single-cell tools for immune monitoring after allogeneic hematopoietic stem cell transplantation (HSCT). Single-cell transcriptomics provides insight into cell identities and functional states. Through B cell and T cell receptor (BCR, TCR) sequencing and deconvolution of donor- and recipient-derived cells, and leveraging expressed germline single nucleotide polymorphisms (SNPs) or mitochondrial DNA mutations, processes such as immune reconstitution, graft-versus-leukemia (GvL) effects and graft-versus-host disease (GvHD) can be dissected at high resolution.

One of the most active areas of immune surveillance has been the study of immune reconstitution after allogeneic stem cell graft infusion and the identification of circulating immune cell populations that associate with graft-versus-leukemia (GvL) effects or graft-versus-host-disease (GvHD) using flow cytometry^57,58^ or mass cytometry.^59,60^ Single-cell genomics studies are now deepening our understanding of these processes, as they additionally resolve transcriptional states, immune receptor repertoires and single-cell donor/recipient origin^61^ of the involved immune cells. Further, single-cell studies are starting to shed light on immune cell dynamics and donor immune contributions in GvHD target tissues, such as skin or the gastrointestinal (GI) tract, which have previously been far less studied, due to technical limitations, such as the inability to distinguish donor- from recipient-derived single cells (**Fig. 2**).^62^

### Immune reconstitution after transplant

Fundamental groundwork for a more complete understanding of post-HSCT immune reconstitution has been laid by Huo *et al,.* who performed scRNA-seq on serially collected bone marrow and peripheral blood samples of 10 patients undergoing transplant for aplastic anemia.^63^ By sequencing FACS-sorted CD34^+^ cells, they documented a proliferative burst of hematopoietic stem cells with skewing towards megakaryocyte-erythroid progenitors, in the first week after graft infusion, followed by a gradual normalization of transcriptional states to a physiologic output of progenitor differentiation until day 30, that was similar across all 10 study subjects. In contrast, scRNA-seq of unsorted populations demonstrated the heterogeneity of circulating immune cell reconstitution following day 30, with large differences in the ratios of monocytes, B and T cells. Another study on immune reconstitution post-HSCT by Obermayer *et al.* monitored T cell reconstitution and TCR repertoires in 5 patients undergoing myeloablative transplant, and found persistence of expanded donor-derived CD8^+^ effector memory T cell clones following graft infusion into the host.^64^ Finally, Luo *et al.* have explored the heterogeneous landscape of regulatory T cells (Tregs), and compared their transcriptional states in the post-transplant context with those from healthy donors, which revealed both shared and distinct gene expression profiles.^65^ Together, these studies confirm previously known dynamics of immune reconstitution following allogeneic HSCT, provide refined insights into transcriptional states and TCR repertoires and establish a framework for future scRNA-seq analyses on post-transplant immune reconstitution.

### Graft-versus-host disease

In contrast to the ready availability of circulating immune cells, technical hurdles in the past have made the study of GvHD in primary human tissue biopsies much more challenging than in peripheral blood. This was due to the lack of high-throughput approaches to distinguish donor from recipient-derived single cells. Single-cell transcriptomics solves several challenges and now enables to address questions such as i) the contribution of donor-derived immunity to tissue-resident memory populations, ii) the persistence of host-derived immunity in GvHD target tissues and, iii) differences in the phenotypes of tissue-resident memory compared to circulating immune cells. This advancement is highly relevant for understanding the mechanisms driving off-target alloreactivity since the study of tissue-resident T cells, which vastly (>10x) outnumber circulating T cells,^66^ requires investigation of primary human tissue biopsies.

In the first scRNA-seq study of post-transplant tissue-resident T cells, Strobl *et al.* systematically dissected tissue chimerism and the phenotypes of skin T cells based on a dedicated longitudinal sample collection of tissue biopsies and matched peripheral blood samples before and at multiple time points post-HSCT.^67^ With an analysis that leveraged expression of single nucleotide polymorphisms^68^ and *de-novo* reconstruction of TCR sequences,^69^ they demonstrated the co-existence of donor- and recipient-derived T cells in two patients on day 14 after the transplant. They extended this analysis further and provided examples of persistent high recipient T cell chimerism in skin biopsies for up to ten years post-HSCT using X/Y fluorescence *in situ* hybridization (FISH).

Using scRNA-seq, Almeida *et al.* have confirmed the longevity of host-derived skin-resident T cells following allogeneic transplant with myeloablative conditioning (MAC) despite complete donor T cell chimerism in the blood in two more cases, and demonstrated that host tissue-resident T cells circulated in peripheral blood after transplant with retained expression of tissue-specific gene expression profiles and tissue-residency markers such as *BLIMP1* and *LGALS3*.^70^ However, these observations were not identified in all 26 patients, as only 6 had incomplete skin donor chimerism. Further, the authors of that work did not find evidence of host skin-resident T cells as drivers of skin GvHD, as suggested by other studies.^62,67^

A study by Jarosch *et al.* characterized gastrointestinal (GI) biopsies via scRNA-seq combined with scTCR-seq and found an increase in Tregs and clonally expanded CD8^+^ T cells to be associated with severe acute GvHD. The clonally expanded CD8^+^ T cells were shared across different GI biopsies from the same individuals, suggesting they are drivers of GvHD. Deorphanizing their antigen specificity would be highly informative for a deeper understanding of the mechanisms underlying GI GvHD and alloreactivity in general.

Together, these studies provide first glimpses into the very incompletely understood phenotypes and TCR repertoires of tissue-resident memory T cells in GvHD. Much remains to be explored, such as the exact trajectory of alloreactive T cells from graft infusion into target tissues or whether their phenotypes and TCR repertoires differ across tissue compartments. The latter question has recently been addressed by DeWolf *et al.* in a systematic study of TCR repertoires across multiple different tissue sites.^71^ The authors identified anatomically defined TCR repertoires and motifs that only partially overlapped with circulating TCR repertoires, underscoring the importance of the study of tissue-resident memory T cells. Besides providing a deeper understanding of T cells, scRNA-seq studies of tissue biopsies should also investigate the role of other cell types in the pathogenesis of GvHD, such as B cells, for which Poe *et al.* have provided a baseline by defining their transcriptional profiles in peripheral blood and bone marrow.^72^ The possibilities of spatial transcriptomics in combination with the detection of immune cell receptor sequences^73,74^ will enable the mapping of alloreactive cells to their cellular targets, and further refine our understanding of GvHD.

### Graft-versus-leukemia effects

Despite decade-long research on GvL effects, it still remains incompletely understood what cellular mechanisms determine protective alloreactivity against disease recurrence, and how they fail when relapse occurs.^75^ Single-cell studies are providing opportunities to address these questions from a fresh angle while leveraging deep sample archives. Despite practice changes in treatments, such as the introduction of tyrosine-kinase inhibitors for *BCR::ABL1^+^* in chronic myeloid leukemia (CML), or Bruton tyrosine kinase (BTK) and BCL2 inhibitors for chronic lymphocytic leukemia (CLL), these longitudinal sample collections remain highly relevant for translational immunologic research.^76^ The fact that donor selection, conditioning regimens and immunosuppression are precisely defined, makes allogeneic transplantation an attractive clinical model for immunotherapy. As such, samples from CML and CLL cases pre- and post-transplant provide opportunities to dissect general mechanisms of GvL that can serve as a framework for improving transplant outcomes for other blood malignancies and may reveal general concepts applicable to other immunotherapies.

In one study, Bachireddy *et al.* investigated resistance mechanisms to GvL by studying matched CLL relapse samples before and following reduced intensity conditioning (RIC)-HSCT using whole-exome sequencing (WES) and scRNA-seq, focusing on genetic and transcriptional changes in cases with early versus those with late relapse post-HSCT. While early post-transplant relapse cases demonstrated genetic and transcriptional stability, late relapse cases were characterized by clonal evolution at the genetic and transcriptional level, likely due to a more sustained immune pressure by GvL.^77^ These GvL-induced changes were heterogeneous and suggest diverging immune escape mechanisms.

In a second mechanistic study on GvL, Bachireddy *et al.* tracked the dynamics of T cell subsets in 6 responders and 6 non-responders to CD8^+^ T cell-depleted DLI for relapsed CML post-HSCT using scRNA-seq and bulk assay for transposase accessible chromatin with high-throughput sequencing (ATAC-seq). Consistent with a prior study on DLI in CML,^78^ they observed enrichment of exhausted T cells in responders compared to non-responders prior to DLI. Integration of scRNA-seq with bulk ATAC-seq data demonstrated the role of transcription factor master regulators such as TOX in T-cell exhaustion. Infusion of DLI led to a contraction of exhausted T cell clusters, but this was largely driven by T cell clones which were detectable before DLI and not those that were uniquely provided by the DLI product, suggesting a mechanism of immunologic support rather than direct provision of CML-specific effector T cells.

These results contrast with analyses on the determinants of successful DLI in acute myeloid leukemia (AML), where Maurer *et al.* have observed the expansion of cytotoxic CD8^+^ T cells in concert with naïve B and other cell populations after infusion, suggesting diverging underlying mechanisms.^79^ Non-response was associated with increased expression of *KLRG1* and *TIGIT*, which is similar to another recent study that identified TIGIT as an important indicator of post-transplant AML relapse.^59,80^ In a study of post-transplant relapse of pediatric AML, Shahid *et al.* found downregulation of HLA class II expression,^81^ confirming observations from adult AML.^24,25^

## Immune checkpoint blockade in AML relapse

**Figure 3.**
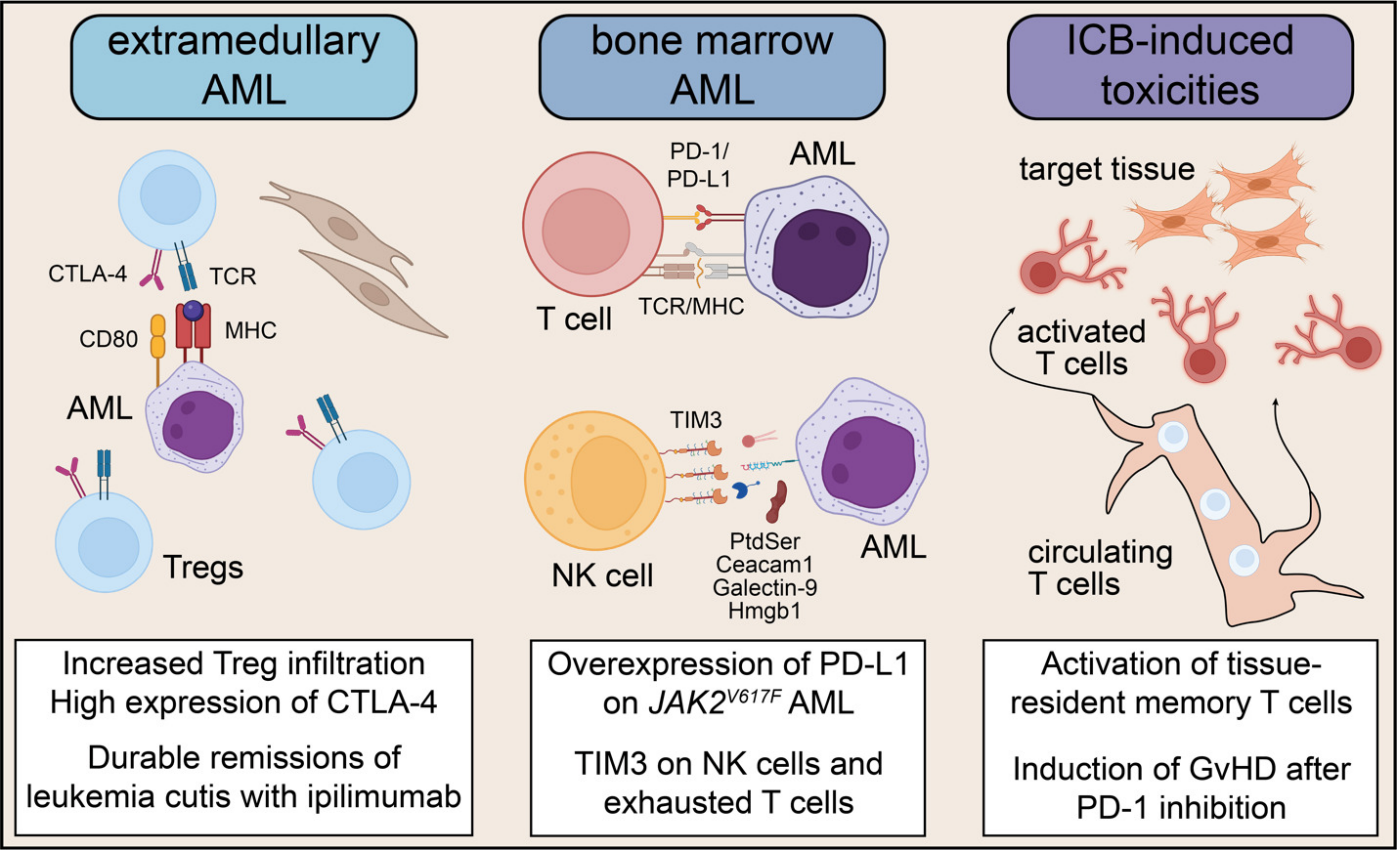
Mechanisms of immune checkpoint blockade activity in acute myeloid leukemia (AML). Ipilimumab has shown durable remissions in extramedullary acute myeloid leukemia (eAML) with skin manifestation (leukemia cutis) likely due to high infiltration of Tregs that express CTLA-4. In bone marrow, inhibition of PD-1 is rationalized by overexpression of PD-L1 in JAK2^V617F^-mutated AML. TIM3 is expressed by natural killer (NK) and exhausted T cells, providing a mechanistic base for TIM3 inhibition. ICB-induced off-target toxicity is associated with activation of effector memory T cells in peripheral tissues. This has limited the use of PD-1 blockade in the post-transplant setting due to intolerable rates of graft-versus-host disease (GvHD).

Myelodysplastic syndrome (MDS) and AML are currently the most frequent indications for allogeneic HSCT in adult hematology, which can improve long-term survival in high-risk cases.^82,83^ However, relapse is still frequent and, therefore, a distinct clinical need exists for strategies that improve anti-leukemic immune responses for AML/MDS. Early experiences with immune checkpoint blockade (ICB) in AML/MDS, such as durable remissions in post-transplant leukemia cutis relapse cases following CTLA-4 blockade (ipilimumab)^84,85^ or favorable associations of pretreatment T cell infiltration with clinical activity of PD-1 blockade (nivolumab) in combination with azacytidine^86^ generated a lot of interest to utilize ICB to address post-transplant and transplant-naïve AML relapse (**Fig. 3**).

This was further supported by murine data that demonstrated overexpression of PD-L1 in *JAK2^V617F^*-mutated myeloproliferative neoplasms (MPN) as an actionable immune escape mechanism.^87^ Penter *et al.* dissected an index case of a secondary *JAK2^V617F^*-mutated AML relapse post-HSCT who received nivolumab based on this rationale, using a multi-omics single-cell approach.^88^ The authors observed an expansion of CD4^+^ T cells after nivolumab infusion that was paralleled by the contraction of a megakaryocytic leukemic cell population characterized by *PD-L1* expression. This megakaryocytic population later recovered at the time of AML progression, when the CD4^+^ T cell population contracted again, consistent with immune evasion of leukemia cells following an activated CD4^+^ T cell GvL response. Tregs progressively expanded within the CD4^+^ T cell compartment during PD-1 inhibition, which may be a potential counter mechanism after PD-1 inhibition.

Although later clinical results do not support ICB as a generally effective immunotherapy in myeloid disease,^89–92^ several clinical studies that tested this approach have been accompanied by single-cell sequencing-based immune monitoring efforts. These have provided instructive vignettes on tumor immunology in AML and have been able to explore more index cases with exceptional responses to ICB, providing starting points for future investigations in the development of immunotherapies for AML/MDS.

### CTLA-4

The observation of durable remissions in post-transplant leukemia cutis relapse following ipilimumab infusion led to the ETCTN/CTEP 10026 study, which tested the hypothesis that an alloreactive environment would enhance clinical activity of ICB. Unexpectedly, the overall response rate to combined decitabine and ipilimumab on ETCTN 10026, which recruited mostly bone marrow-involved AML/MDS, was higher in the transplant-naïve compared to the post-transplant arm, and responses were short-lived.^93^ Large-scale scRNA-seq analyses of 64 bone marrow samples from 18 patients showed that CTLA-4 blockade increased the percentage of Tregs, likely as a compensatory mechanism to ipilimumab infusion, and demonstrated that the short duration of responses was associated with insufficient clearing of leukemic clones from progenitor cells.

Durable remissions observed with CTLA-4 blockade in extramedullary AML, and the higher activity of ipilimumab in cancers such as metastatic melanoma, prompted additional investigations into immunologic differences between bone marrow-involved AML, leukemia cutis and solid malignancies. Compared to scRNA-seq data of T cells from solid malignancies, AML bone marrow cell profiles had much fewer features of T cell exhaustion. A comparative analysis by bulk RNA-seq revealed higher infiltration of CTLA-4^+^ T cells in extramedullary versus bone marrow-involved AML.^94^ Interestingly, one exceptional post-transplant responder with bone marrow disease had an ongoing response for more than 3 years after combined decitabine and ipilimumab treatment. In that case, the pretreatment bone marrow was heavily infiltrated by CTLA-4^+^ and PD-1^+^ T cells, suggesting a preexisting GvL response whose clinical activity was further enhanced by ipilimumab. Overall, this study indicates that extramedullary AML associates with immunologic features that confer susceptibility to immunotherapy and provides opportunities for therapeutic targeting that should be further investigated. One such potential research direction has been proposed by Koedijk *et al.*, who have used spatial analyses to demonstrate the existence of large T cell networks that associate with response to CTLA-4 blockade, and could represent a marker of susceptibility to immunotherapy.^95^

### PD-1

Several studies with diverging insights illustrate that the mechanisms of response to PD-1 inhibition in AML seem to be heterogeneous.

Apostolova *et al.* reported signatures of higher T cell activation and lower senescence in CD8^+^ T cells that distinguished responders from non-responders to combined decitabine/azacytidine and nivolumab treatment.^96^ In one notable study participant, who maintained an incomplete remission (CRi) for 11 months after discontinuing nivolumab, scRNA-seq revealed signs of distinct immune activation with donor-derived monocytes which had high expression of HLA class II molecules. These observations provide evidence of nivolumab-induced immune activation as the driver for the observed reinvigorated GvL activity.

In contrast, Goswami and Gui *et al.* observed clonal expansion of activated effector T cells with expression of markers such as HLA-DR, PD-1 and TIM-3 in three non-responders who experienced immune toxicities (central diabetes insipidus and hypothyroidism) after combined treatment with decitabine and the PD-1 blocking antibody pembrolizumab.^97^ This suggests these expanding T cell clones are involved in the reported adverse events and opens up the question about their antigen specificity, given that hypothyroidism is a frequent toxicity of therapeutic PD-1 blockade. Remarkably, unlike in study participants with immune-related adverse events, the authors failed to observe T-cell clonality or phenotype changes in two responders, illustrating difficulties in disentangling decitabine- from nivolumab-induced therapeutic effects.

In another study, Abbas *et al.* investigated 22 serial samples of 8 study participants who received azacytidine and nivolumab. AML cases were characterized by higher clonal T cell expansion compared to two healthy donors. They displayed diverging dynamics of changes in clonal expansion between responders and non-responders during PD-1 inhibition. In one notable responder case, loss of chromosome 7 developed following response to nivolumab-based treatment and could have represented an immune resistance mechanism in that case.

Together, these analyses represent illustrative examples of how single-cell genomics of samples obtained from clinical studies can provide deep and integrative immune and AML monitoring.

### TIM-3

TIM-3 is a third immune checkpoint molecule whose inhibition is being investigated in AML/MDS^98,99^ with opportunities for correlative immunologic studies. Huuhtanen *et al.* have presented initial analyses into responses to TIM-3 inhibition in AML.^100^ Their systematic reanalysis of 500,000 scRNA-seq profiles across 160 bone marrow aspirates showed higher TIM-3 expression levels in activated T cells and hematopoietic stem cells (HSCs) in individuals with blood malignancies compared to healthy donors, underscoring a rationale for therapeutic TIM-3 inhibition. Interestingly, NK cells also have high TIM-3 expression, suggesting that they may play an important role in the clinical activity of TIM-3 inhibitors, which may be especially relevant for the early post-transplant context, due to the faster NK immune cell reconstitution compared to T cells.^58^

## CAR T cell therapy

One of the most significant developments in immunotherapies for blood malignancies has undoubtedly been the introduction of CAR T cell therapy directed against the B cell lineage markers CD19 and B-cell maturation antigen (BCMA).^101,102^ The wide expression of these markers on malignant B cells that are the foundation for fast and deep remissions have enabled the impressive clinical success of this therapeutic strategy. Nevertheless, relapse still occurs frequently even with CAR T cell therapy, and only about 50% of patients achieve a sustained complete remission. This has motivated many single-cell genomics-based immune monitoring studies to investigate the determinants of successful CAR T cell therapy and mechanisms driving resistance. Finally, although generally manageable, on-target off-tumor long-term toxicities following CAR T cell infusion (i.e., sustained B cell aplasia) are another important area of research, and studies identifying molecular features of adverse events, such as acute neurotoxicity which often occurs within days of CAR T cell infusion, are now emerging.

### CD19

**Figure 4.**
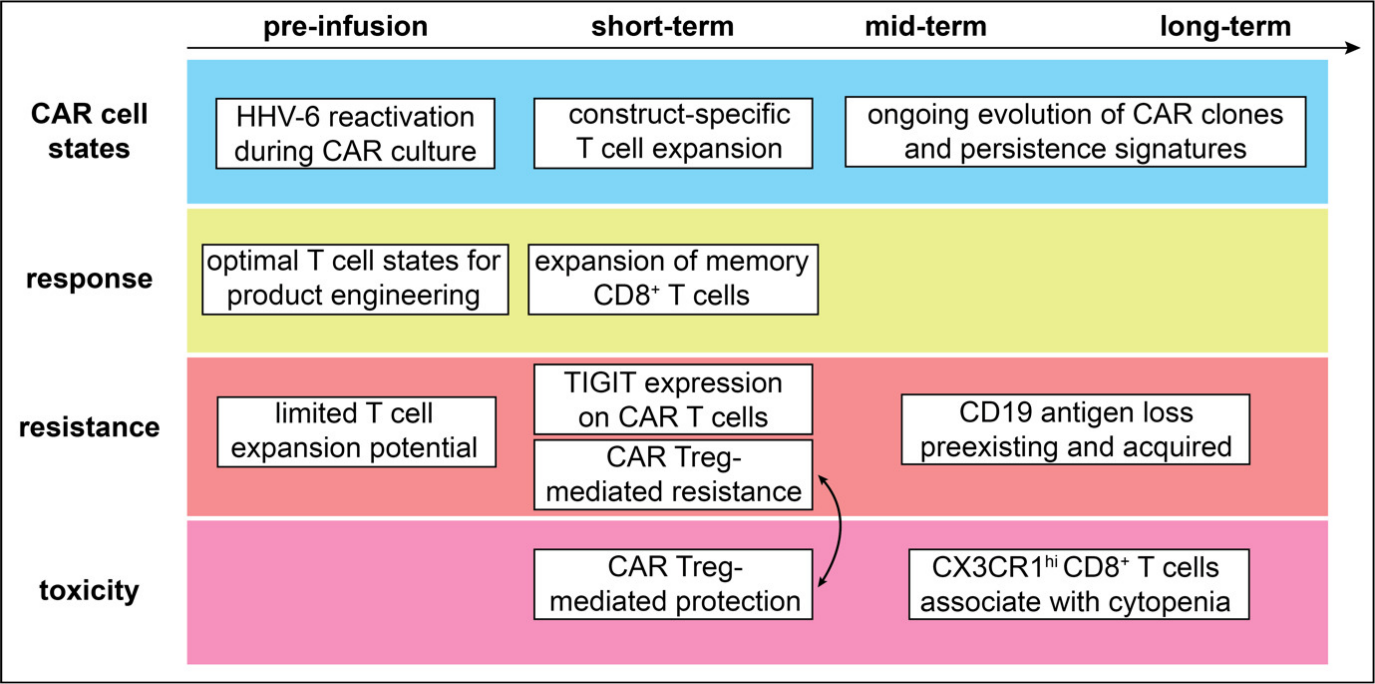
Examples of mechanistic insights on CD19-directed CAR T cell therapy gained through single-cell RNA sequencing studies. Single-cell studies were focused on T cell states of CAR infusion products (pre-infusion), short-term effects that lead to response, resistance, or occurrence of immune-related toxicities in the immediate days after infusion (short-term) and mid- or long-term effects of CAR T cell therapy.

CAR T cell therapy against CD19 is certainly the model for a broadly applicable cellular therapy with a profound impact on treatment outcomes in many B cell malignancies. The success of this therapy has also led to large single-cell sequencing immune monitoring studies to define its optimal use. These studies focus on molecular and cellular features that are associated with early aspects of CD19 CAR T cell therapy (i.e., manufacturing processes and cellular dynamics immediately following infusion), medium- or long-term aspects (mechanisms mediating disease relapse, persistence of CAR T cells) or adverse immune toxicities. Together, they have provided us with precise insights into the phenotypes of CAR T cells that constitute starting points for improvements in the development of this cellular therapy (**Fig. 4**).

Several studies have focused on transcriptional profiles of infusion products as biomarkers for response. For example, Deng *et al.* found that response to axicabtagene ciloleucel (axi-cel) in large B cell lymphomas (LBCLs) was associated with fewer exhausted T cells and higher frequencies of memory T cells.^103^ This was consistent with results from a study by Chen *et al.,* who found that apheresis products with higher proportions of naïve, stem cell memory and central memory T cells were associated with CAR T cell persistence beyond 6 months ,^104^ and with results from Saren *et al.,* who found polyfunctional effector-like CD8^+^ T cells to associate with clinical response.^105^

Finally, Bai *et al.* identified a deficiency in T helper 2 function that was associated with relapse in a study of 12 CAR infusion products.^106^ Together, these results indicate that manufacturing of CAR T cell infusion products should take into consideration apheresed T cell states, for example by enriching for naïve and other less differentiated subsets. They also suggest that allogeneic CAR T cell products from healthy donors may be advantageous for higher clinical efficacy.^107^

By screening published scRNA-seq data of CAR T cell infusion products, Lareau *et al.* made the remarkable observation that the manufacturing process likely leads to reactivation of HHV-6 in rare cases (28 out of 819,321 screened cells).^108^ This constitutes a potential source for fatal CNS HHV-6 infections that have been observed after CAR T cell therapy.^109–112^

Going beyond the CAR T cell manufacturing process, Sheih *et al.* performed short-term tracking of CAR T cells from infusion products and peripheral blood up to 112 days after infusion.^113^ They observed a large polyclonal expansion of CD8^+^ CAR T cells within the first 10 days post-infusion and a progressive change in transcriptional state. Initially, CAR T cells displayed gene expression profile with features driven by the high metabolic activity manufacturing process that changed over time to a less activated transcriptomic state.

Dissection of transcriptional states in CAR infusion products and their short-term longitudinal dynamics after infusion were further refined by Haradhvala, Leick & Maurer *et al.* who addressed the question of whether axi-cel and tisagenlecleucel (tisa-cel) have different mechanisms of response and resistance. They performed scRNA-seq on both CAR products, matched circulating CAR T cells at day 7 after infusion, and found marked differences in their composition and post-infusion cellular dynamics.^114^ While axi-cel infusion products contained variable amounts of CD8^+^ T cells, tisa-cel contained very few CD8^+^ T cells that rapidly expanded by day 7, driven by the most expanded clones in the infusion product. On the other hand, the proliferation of tisa-cel CD4^+^ CAR T cells very quickly stopped after infusion. Transcriptional profiles of CAR T cells differed by product, such that, for example, CD28 signaling of axi-cel associated with higher *PD-1* expression at day 7.

Both studies found that individual CAR T cell clones identified by their native TCR sequence changed in frequency and phenotype over time, illustrating that the population of infused CAR T cells continues to evolve, likely due to sustained interaction with their target antigen, either from recovering physiologic B cell lymphopoiesis or minimal residual disease.

Further evidence for the continued evolution of CAR T cells, but also their remarkable *in-vivo* longevity, has been documented by a scRNA-seq study that showed the persistence of proliferative CD4^+^ CAR T cells for more than 10 years in two patients with sustained CLL remission and B cell aplasia.^115^ In a second scRNA-seq study on the long-term persistence of CAR T cells in 15 pediatric ALL patients, Anderson *et al.* observed that CAR T populations were increasingly dominated by CD4/CD8 double-negative cells more than one year after infusion. They were able to define a signature of long-term CAR T cell persistence including expression of the immune checkpoint molecule *TIGIT* or the effector molecule *GZMK*. Through analysis of native TCR sequences expressed by CAR T cells, they could demonstrate that CAR T cell populations remain highly polyclonal even years after infusion.^116^

Multiple scRNA-seq studies have found mechanisms of non-response and relapse after CD19 CAR therapy, which can be categorized as a result of immune escape by tumor cells (i.e., loss of the target antigen CD19) or suboptimal CAR T cell function.

CD19-negative disease relapse after CD19-directed CAR T cell therapy is frequent and one of the clinically most relevant resistance mechanisms, as it renders re-exposure to CAR T cells futile.^110,117^ Multiple mechanisms of CD19 downregulation have been described.^118–122^ However, it is not fully understood whether tumor cells with low CD19 expression generally exist prior to CAR T cell infusion or whether the loss of CD19 expression develops *de-novo* under therapeutic pressure. Im *et al.* investigated this question through *in-vitro* analyses and found that interactions between CAR T cells and tumor cells lead to a sustained downregulation of surface CD19 expression through internalization, arguing against preexisting CD19-negative tumor cells.^123^ In contrast, Rabilloud *et al.* dissected one case of CD19-negative ALL relapse after CAR with the help of scRNA-seq, and could show that this was due to the expression of a non-functional CD19 isoform in ALL cells, which were detectable even prior to CAR T cell therapy. This suggests that screening for CD19-negative ALL cells prior to infusion may help to anticipate disease relapse in at least a faction of cases.^124^

Besides the loss of the target antigen CD19, several immune resistance mechanisms to CD19 CAR T cell therapy have been found. The study by Haradhvala *et al.* identified CAR Tregs which were detectable in both infusion products (axi-cel and tisa-cel) and associated with non-response and relapse to CD19 CAR T cell therapy. A second study by Good *et al.* linked CAR Tregs to therapeutic resistance through inhibition of CD8^+^ CAR T cell expansion (axi-cel) after infusion in 32 cases of large B cell lymphoma (LBCL).^125^ This finding was consistent across both studies, and suggests a potential strategy for improvement of the CAR T cell manufacturing process through the depletion of CAR Tregs from infusion products.

Increased expression of the immune checkpoint molecule *TIGIT* on circulating CAR T cells is another resistance mechanism identified using two scRNA-seq studies.^126,127^ Jackson *et al.* immunophenotyped 4-1BB CAR T cells from 14 manufacturing products and from 27 peripheral blood samples 14 to 30 days after infusion in 13 non-Hodgkin lymphoma (NHL) cases.^127^ They found circulating CAR T cells to express higher *TIGIT* levels in non-responders, and could validate the relevance of this finding in an *in-vivo* model in which TIGIT blockade led to longer survival of mice challenged with lymphoma cells and CAR T cells.

In contrast, by tracking SJCAR19 CAR T cells longitudinally in 16 pediatric B-acute lymphoblastic leukemia (ALL) cases before and up to 6 months after infusion using their native TCR sequences as clonal barcodes, Wilson *et al.* found that *TIGIT* expressing effector precursor CD8^+^ CAR T cells constitute a subpopulation within infusion products that has superior functionality and is less prone to acquire an exhausted phenotype.^128^

These apparent discrepancies may relate to the different CAR T cell products investigated, but also inherent differences of the pediatric or adult setting, including prior therapies. This illustrates an opportunity for integrative data reanalysis across the different studies to understand better commonalities and differences of CD19 CAR T cell therapies in different contexts.

Finally, single-cell studies are starting to identify factors associated with adverse events after CAR T cell infusion, such as neurotoxicity, a potentially life-threatening adverse event that occurs in a substantial percentage of cases (38-77%)^129,130^ or prolonged cytopenias. The study by Good *et al.* found CAR Tregs to be protective against the occurrence of immune effector cell-associated neurotoxicity syndrome (ICANS).^125^ The fact that CAR Tregs were also associated with disease progression indicates that future studies could focus on identifying an optimal level of their frequency in CAR T cell products to reduce ICANS while retaining disease control, similar to efforts of fine-tuning GvL and GvHD after transplant.

Strati *et al.* identified clonally expanded CX3CR1^hi^ cytotoxic T cells associated with prolonged cytopenia after axi-cel CAR T cell infusion in a study of 16 LBCL cases.^131^ These T cells also included clones that did not express a CAR transcript, raising the possibility of cross-talk between CAR and non-CAR T cell populations. Deorphanizing TCR antigen specificities of scRNA-seq studies that identify associations of immune toxicities with expanded T cell populations would be very valuable.

### BCMA

Targeting BCMA for multiple myeloma is a second CAR T cell therapy that has gained a lot of traction in recent years,^132,133^ with emerging single-cell genomics studies starting to better define determinants of response and resistance to BCMA CAR.

Similar to CD19^-^ relapse, targeting BCMA via CAR T cell therapy can lead to loss of the target antigen. Two scRNA-seq studies have investigated the underlying genetics and showed this to arise due to a biallelic loss of BCMA.^134,135^ Other mechanisms that lead to BCMA loss include missense and frameshift mutations.^136^ Although heterozygous loss of BCMA is frequent in multiple myeloma before CAR T cell infusion, BCMA loss as a resistance-driving mechanism is nevertheless less common than CD19^-^ relapse after CD19 CAR.^137^ Single-cell RNA sequencing studies are also emerging to define immune-related toxicity after BCMA CAR T cell infusion, for example, in a case of sarcoidosis-like disease^138^ and cellular dynamics associated with response and resistance in individual cases.^139,140^ This includes a study on immune-cell correlates with response to BCMA CAR by Dhodapkar *et al.* that provides the first glimpses into the long-term effects of this therapeutic approach.^141^

In the coming years, a wealth of studies will undoubtedly be forthcoming that will investigate immune cell correlates of response, resistance, and toxicities to BCMA CAR. The body of work in the field of CD19-directed CAR T cell therapy will be a useful framework for this.

## Tracking cancer cell evolution using natural genetic barcodes

**Figure 5.**
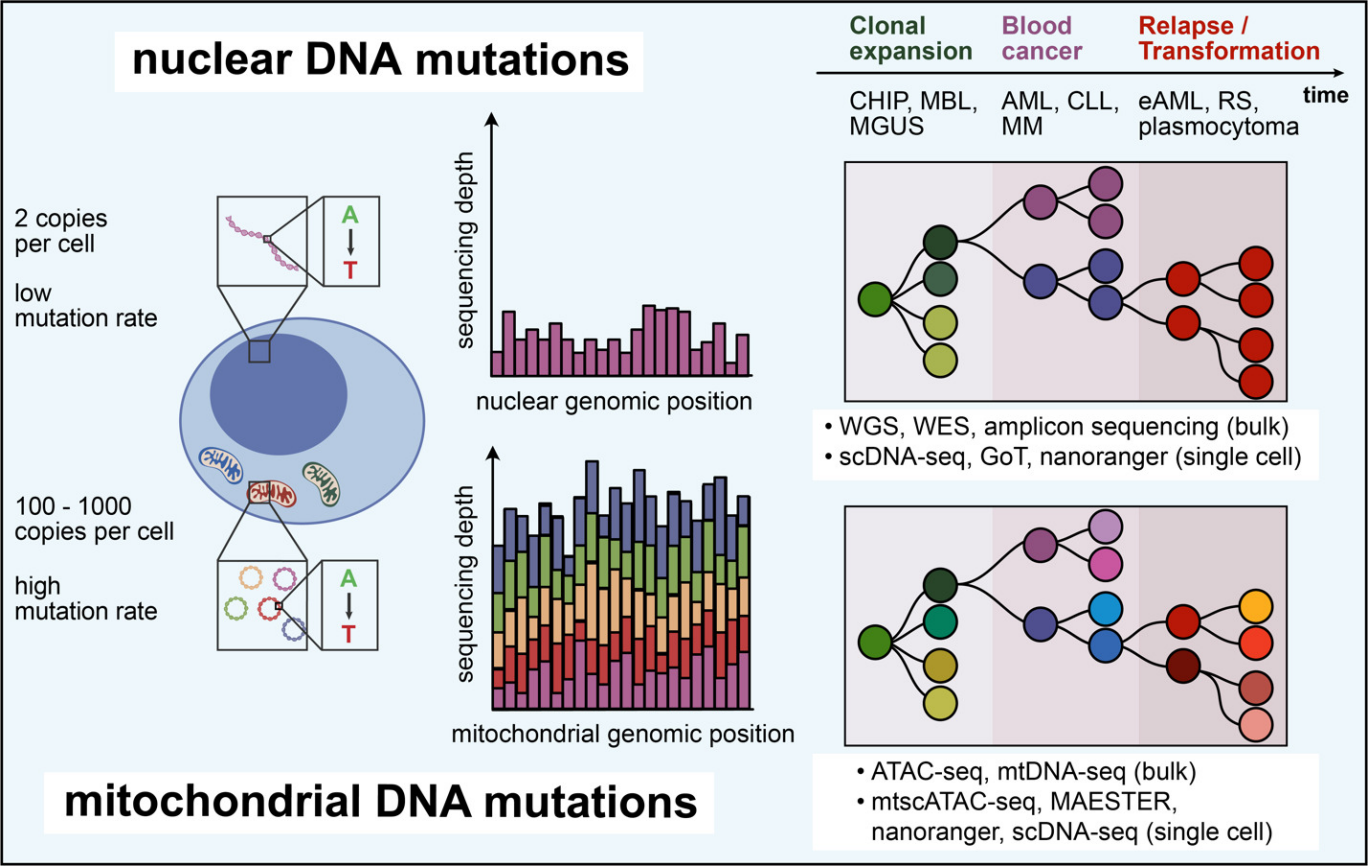
Tracking of clonal evolution using somatic nuclear and mitochondrial DNA mutations. Somatic nuclear mutations are an established approach for tracking clonal evolution from premalignant clonal expansion to blood cancer and to transformed disease. In comparison to mitochondrial DNA (mtDNA), nuclear genomic DNA has a lower mutation rate and generally only 2 copies per cell, which makes attaining sufficient coverage for variant calling with single-cell sequencing approaches challenging. The high mutation rate of mtDNA and the better genomic capture at single-cell resolution potentially enable the higher-resolution tracking of clonal hierarchies, while the integration of both approaches is further maturing. CHIP – clonal hematopoiesis of indeterminate potential, MBL – monoclonal B lymphocytosis, MGUS – monoclonal gammopathy of undetermined significance, AML – acute myeloid leukemia, CLL – chronic lymphocytic leukemia, MM – multiple myeloma, RS – Richter’s syndrome, eAML – extramedullary AML WGS – whole-genome sequencing, WES – whole exome sequencing scDNA-seq – single-cell DNA sequencing

Besides pioneering immunotherapy and methods for monitoring immune responses, blood malignancies have been models for the longitudinal study of genetic evolution that leads to malignant transformation, underpins acquisition resistance following immunotherapeutic interventions and drives secondary transformation from low- to high-grade disease states (**Fig. 5**). Single-cell sequencing approaches have become valuable tools for dissection of these processes, which are transforming our understanding of clonal relationships between physiologic and malignant hematopoiesis. These studies herald the adoption of single-cell sequencing as a clinical tool for monitoring therapeutic responses or acquisition of resistance at high resolution.

### Somatic nuclear mutations

Amongst all genetic markers that enable tracking blood malignancies, somatic single nucleotide variants (SNVs) represent arguably the best-studied class, due to their high frequency^142^ and ease of detection. SNVs arise from endogenous processes like DNA replication error but also from exogenous causes such as exposure to toxic environmental factors.^143^ Their occurrence is associated with premalignant states such as clonal hematopoiesis of indeterminate potential (CHIP),^144^ monoclonal B cell lymphocytosis (MBL)^145^ or monoclonal gammopathy of undetermined significance (MGUS),^146^ and ultimately development of cancer.

The documentation of the full genetic landscape of blood malignancies in newly diagnosed or relapsed diseases was enabled by whole exome sequencing (WES) and whole genome sequencing (WGS) in combination with systematic blood and bone marrow banking programs. Major fundamental works included large-scale genetic characterizations of MBL/CLL,^147,148^ the genomic classification of AML,^149^ ALL^150^ and B cell lymphomas like MM^151^ or diffuse large-cell lymphoma.^152^ WES has also established the relationships between premalignant states and blood cancer. CLL and AML were found to genetically resemble their precursor conditions,^153–157^ while MM has been characterized to harbor increased genetic complexity compared to MGUS.^158^ These foundational studies have provided major insights into pathogenesis and prognostication in blood malignancies, insights which have profoundly changed their risk stratification and therapeutic approaches. Moreover, they are the starting point for many contemporary single-cell genomics studies.

Longitudinal analyses of matched samples from initial diagnosis and relapse have further established the concept of clonal evolution following therapeutic bottlenecks that give rise to increasingly selected and resistant leukemia clones. Due to the decade-long natural disease history and the numerous consecutive treatments, CLL has been one of the model systems for the study of clonal evolution.^147,159,160^ In AML, longitudinal genetic studies have established diverging patterns of resistance to therapy, including persistence and further linear evolution of a founding clone, or outgrowth of a branch of subclones that replace the founder.^161,162^ These insights have also sparked investigations into the clonal dynamics of malignancies for which tumor cells are more difficult to obtain, such as MM or Hodgkin’s lymphoma^163^ that have documented evolution towards increased genetic complexity following therapeutic bottlenecks.^164,165^ Together, they have motivated deeper elucidation of subclonal heterogeneity at the single-cell level.

With single-cell sequencing (scSeq) experiencing its “breakthrough” in 2018 (https://vis.sciencemag.org/breakthrough2018/finalists/), genetic studies in blood malignancies can now be conducted at truly high resolution. In bulk WGS/WES data, variant allele fractions (VAF) can generally be confidentially called down to 0.1-1% due to amplification or sequencing artifacts that may reach VAFs of 0.1-1%.^166^ A further challenge of bulk sequencing data analysis is the imputation of clonal heterogeneities from VAFs. With scDNA-seq, these boundaries have been profoundly shifted: clonal substructures can be directly observed at the level of individual single cells. Further, malignant clones can be tracked across cell types, which resolves clonal hierarchies between progenitor and differentiated cell compartments. Finally, it is possible to distinguish somatic mutations that are expanded within tumor cells from those in unrelated immune cells without sophisticated sequencing of sorted bulk populations.^167^

The technical improvements of single-cell sequencing have provided direct evidence of clonal competition, but are also enabling the tracking of individual rare malignant cells. For example, an early targeted scDNA-seq study of two AML cases by Pellegrino *et al.* documented the persistence of leukemia cells at the time of remission and their clonal heterogeneity.^168^ Miles *et al.* utilized this approach systematically by sequencing 143 samples from 123 patients based on the detection of 31 recurrent somatic mutations, and tracked co-existing AML subclones back to their origin in MPN.^52^ Morita *et al.* similarly tracked mutational histories in 154 samples from 123 AML cases and provided insight into clonal competition and selection of resistance clones during therapy such as FLT3 inhibition.^20^ The concepts of clonal competition and evolution have also been studied in other blood malignancies including B^150^ and T-ALL,^169,170^ CLL^171^ or MM.^172–174^

Together, these studies showcase the potential of single-cell sequencing for the clinical tracking of the emergence of disease resistance during therapy, including in the setting of MRD. In the context of allogeneic HSCT, the detection of MRD has a particular advantage as SNPs may be used for the identification of donor- and recipient-derived cells, which can further aid in the detection of residual leukemic cell populations.^175^

Going beyond mere identification of malignant cells, multi-omics platforms are increasingly becoming available as technologies to define the transcriptional states of individual leukemia clones. Among the pioneering works are studies by Nam *et al.*^53^ and van Galen *et al.*^54^ which both provided approaches for the integrated detection of somatic mutations and gene expression profiles in a high-throughput manner (“genotyping of transcriptomes” – GoT). These studies could demonstrate that MPN clones harboring *CALR* mutations gained a selective advantage over other clones and exhibited upregulated NF-κB signaling, tracked six differentiation states of AML from HSC-like to dendritic-like cells, and performed differential gene expression analyses between normal and malignant hematopoiesis. Central challenges of GoT are the dependence on sufficient expression levels of targeted genes and the genomic location of many somatic mutations in the middle of their respective transcripts, which makes identification with short-read sequencing difficult and leads to many cells without genotyping information. Several solutions have been proposed to improve the success rate of defining the transcriptional states of genetically defined leukemia cells. Petti *et al.* have demonstrated that, by analyzing private, non-recurrent somatic mutations identified by parallel WGS, it is possible to genotype a large number of scRNA-seq profiles, even without additional targeted sequencing.^176^ Another possibility is the employment of long-read sequencing, which enables the detection of mutations distant from the ends of a transcript. The “nanoranger” protocol leverages the much-improved sequencing accuracy of Oxford Nanopore sequencing.^177^ Finally, a potential solution is the combination of scDNA-seq with scRNA-seq, as demonstrated by Rodriguez-Meira *et al.*,^178^ albeit at the cost of a more complex workflow.

Regarding translational applications, all these approaches have in common the potential to perform leukemia tracking in combination with sensitive detection of resistance-mediating gene expression profile changes. As the throughput of single-cell sequencing increases, this may be used clinically to track the emergence of immune escape after effective immunotherapy before an overt relapse. An example could be early detection of CD19^neg^ leukemia relapse after CAR T cell therapy at the time of MRD.

### Mitochondrial DNA mutations

Although the focus of single-cell lineage-tracing efforts in blood malignancies has been on somatic nuclear DNA mutations, in recent years mitochondrial DNA (mtDNA) mutations have gained increasing attention as a natural barcoding system that additionally enables to establish clonal relationships with healthy tissues without the need for prior synthetic barcoding.^50,179–181^ In contrast to somatic nuclear mutations, mtDNA mutations occur frequently in almost all cells and thus potentially greatly expand opportunities for lineage tracing. Given its cell division-independent replication, mtDNA has a mutation rate that is 10 – 100x higher than nuclear genomic DNA.^182^ Its circular structure, short total length of approximately 16.6 kb, and high copy number of 100 – 1000 per cell^183–185^ make mtDNA ideal for high-coverage single-cell readouts and confident *de novo* variant calling.

Due to the unique non-chromatinized state of mtDNA, its mutations can be well-detected using a modified single-cell ATAC-sequencing protocol (mtscATAC-seq), which provides information on genetic lineage and cell states.^180^ The first demonstration of the potential of mtDNA mutation analysis with mtscATAC-seq to dissect longitudinal clonal evolution was provided in CLL, where Penter *et al.* used mtDNA mutational profiles to dissect the effects of chemoimmunotherapy or allogeneic HSCT on leukemia cell subpopulation. Changes in mtDNA mutations correlated with these therapeutic bottlenecks and subclones with distinct chromatin accessibility profiles could be identified.^181^ This opens up the prospect of defining subclones below the level of recurrent somatic mutations and, thus, potentially finer dissection of heterogeneous leukemia populations. Poos *et al.* have provided another demonstration of this concept, using mitochondrial DNA mutations to track evolving subclones in MM throughout therapy.^186^

In the non-transformed context, mtDNA mutations are also useful markers of clonal ancestries that enable to address questions of tissue regeneration, for which hepatocytes are a model system due to their unique ability to dedifferentiate, expand, and redifferentiate.^187,188^ Passman *et al*. have demonstrated this approach by tracking spatially restricted clonal expansion among hepatocytes based on detecting mtDNA mutations.^189^ These results indicate that mtDNA mutations may also have great potential for the study of premalignant states that precede blood malignancies, for example, by dissecting clonal hematopoiesis below the level of individual driver mutations, as shown by Miller *et al.* in one selected case.^190^

One central question in this regard is how mitochondrial and somatic nuclear DNA mutations relate. Studies have addressed this by combining their detection in the same cell.^191^ Velten *et al.* have identified nuclear and mitochondrial single-nucleotide variants (SNVs) from scRNA-seq profiles in the bone marrow of AML.^56^ They could show that both mitochondrial and somatic nuclear DNA mutations provided complementary information for the identification of healthy and malignant hematopoietic clones and were able to distinguish preleukemic from leukemic cells. Developing this approach further, the group developed ‘CloneTracer’, an analytical framework that integrates mtDNA and somatic nuclear mutations based on Bayesian statistics to also correct for dropouts and false positive rates in (sub﻿-﻿)clone identification.^55^ Applying this method enabled the tracking of AML differentiation states, including those in the erythroid compartment, and the definition of aberrant surface marker expression that may enable improved identification of AML based on flow cytometry.

While mtDNA mutations provide novel avenues to the study of clonal evolution in blood malignancies, their unique genetics may come with specific challenges and opportunities to investigate aspects of mitochondrial biology. These include the potential to investigate the horizontal mitochondria transfer between cells, understand mechanisms of negative selection of mtDNA mutations that convey a competitive disadvantage,^192^ quantify the stochastic distribution of mitochondria amongst daughter cells during cell division and convergence of independent mutational events within the confined space of the mitochondrial genome,^193^ which will require further studies that perform side-by-side tracking of somatic nuclear and mtDNA mutations.

## Challenges and outlook

Single-cell sequencing studies of immune and malignant cells in blood malignancies provide deep insights into the heterogeneity of both compartments from a single sample. With the maturation of the underlying assays and the increasing sizes of study cohorts that now match those of many bulk sequencing efforts, single-cell sequencing studies are gradually becoming the workhorse of hematologic translational research. Given the growing range of modalities that can be co-detected in a single cell,^194^ including methods for recording spatial information at high resolution, analyzing these rich single-cell datasets is increasingly a substantial bottleneck. An important future direction will, therefore, be the development of standardized tools that streamline recurrent analytical steps, such as the exploration of immune repertoire data or the analysis of genetic data obtained from single-cell profiles and, perhaps, even analytical approaches that leverage artificial intelligence. Similarly, with the many possibilities of what can be measured, an important question is also what should be measured. For example, while numerous efforts have been made to describe T cell phenotypes in peripheral blood associated with clinical outcomes, focusing on rare circulating antigen-specific or tissue-resident T cells may be of higher yield. Likewise, while single-cell studies of diagnostic or relapsed leukemia samples with high disease burden have documented clonal heterogeneity, it may be rewarding to start focusing on MRD^+^ disease after immunotherapeutic bottlenecks to better understand what enables the persistence of residual leukemia cells.

Finally, as the costs of high-throughput single-cell sequencing are expected to drop in the coming years, and assays are developed that do not depend on sophisticated microfluidics devices,^195^ the prospect of bringing individualized, prospective immune and leukemia single-cell monitoring into the clinic is a real possibility. One example for such a translational application may be an integrated post-transplant monitoring that will provide information on engraftment across the various immune cell compartments, tracking of residual recipient-derived hematopoiesis and, most importantly, detection of any emergence of immune escape variants, such as residual leukemia cells that downregulate HLA presentation or increase their expression of immune checkpoint molecules, all from the same single-cell dataset. Provided a streamlined analytical pipeline can be established, this may be an elegant approach to survey all these aspects in one unified single-cell assay and could meaningfully impact genomics-guided patient care.

### Conflict of Interest

The Broad Institute of MIT and Harvard has filed patent applications relating to the use of technologies for detecting mitochondrial DNA mutations, in which L.S.L. is named as an inventor (US patent applications 17/251,451 and 17/928,696). L.S.L. is a consultant to Cartography Biosciences. The other authors declare no competing financial interests.

### Author Contributions

A.C.R. contributed to the section on tracking clonal evolution and figure 5. L.S.L. reviewed the manuscript. L.P. designed figures and wrote the manuscript.
